# Size Effects of Copper Oxide Nanoparticles on Boosting Soybean Growth via Differentially Modulating Nitrogen Assimilation

**DOI:** 10.3390/nano14090746

**Published:** 2024-04-25

**Authors:** Yaozu Guo, Hao Li, Yi Hao, Heping Shang, Weili Jia, Anqi Liang, Xinxin Xu, Chunyang Li, Chuanxin Ma

**Affiliations:** 1 Key Laboratory for City Cluster Environmental Safety and Green Development of the Ministry of Education, School of Ecology, Environment and Resources, Guangdong University of Technology, Guangzhou 510006, China; 2112124020@mail2.gdut.edu.cn (Y.G.); 2112124044@mail2.gdut.edu.cn (H.L.); haoyi0305@gdut.edu.cn (Y.H.); hshang@gdut.edu.cn (H.S.); 1112324016@mail2.gdut.edu.cn (A.L.); 1112024004@mail2.gdut.edu.cn (X.X.); cyli1969@gdut.edu.cn (C.L.); 2 Guangdong Provincial Key Laboratory of Chemical Pollution and Environmental Safety & MOE Key Laboratory of Theoretical Chemistry of Environment, SCNU Environmental Research Institute, School of Environment, South China Normal University, Guangzhou 510006, China

**Keywords:** soybean, CuO NPs, size effect, nitrogen assimilation, nanofertilizers

## Abstract

Nanoscale agrochemicals have been widely used in sustainable agriculture and may potentially affect the nitrogen fixation process in legume crops. The present study investigated the size-effects of copper oxide nanoparticles (CuO NPs) on nitrogen assimilation in soybean (*G. max* (L.) Merrill) plants, which were treated with different sizes (20 and 50 nm) of CuO NPs at low use doses (1 and 10 mg/kg) for 21 days under greenhouse conditions. The results showed that 50 nm CuO NPs significantly increased the fresh biomass more than 20 nm CuO NPs achieved at 10 mg/kg. The activities of N assimilation-associated enzymes and the contents of nitrogenous compounds, including nitrates, proteins, and amino acids, in soybean tissues were greatly increased across all the CuO NP treatments. The use doses of two sizes of CuO NPs had no impact on the Cu contents in shoots and roots but indeed increased the Cu contents in soils in a dose-dependent fashion. Overall, our findings demonstrated that both 20 and 50 nm CuO NPs could positively alter soybean growth and boost N assimilation, furthering our understanding that the application of nanoscale micro-nutrient-related agrochemicals at an optimal size and dose will greatly contribute to increasing the yield and quality of crops.

## 1. Introduction

Since the 19th century, it has been found that a Bordeaux mixture (CuSO_4_) can prevent plants from bacterial infection. A tremendous number of metals or metal oxides were applied to agriculture production. Nowadays, nanotechnology, especially metal or metal oxide nanoparticles (NPs), provides an efficient way to promote growth and yield [1,2] and resist pests and diseases [3] in crops, as determined by their particle size effects [4]. Thus, in recent years, metal or metal oxide NPs have been commonly used in agriculture as novel growth regulators, nanofertilizers, and nanopesticides [5,6].

Copper (Cu) is one of the essential nutrients for plant growth and development. It is involved in redox reactions of enzymes [7] and participates in hormone signaling pathways, metabolism, and biochemical processes such as photosynthetic electron transfer, mitochondrial respiration, and cell wall metabolism in plants [8]. CuO NPs are among the most common nanoparticles used in industry and agriculture because of their antibacterial activity and bio-availability. The application doses of CuO NPs have positive or negative impacts on plants. For example, CuO NPs at 0.1 mg/L significantly promoted the germination of wheat seeds and subsequent growth of roots [9]; when the use dose was beyond 200 mg/L, CuO NPs inhibited the growth of mung beans and wheat [10]. Therefore, prior to the large-scale application of CuO NPs in agriculture, it is important to find an idealistic use dose to avoid potential damage to plants caused by high doses of CuO NPs [11]. Additionally, plant species is another factor that can determine the doses of CuO NPs. For example, exposure to 150 μg/L CuO NPs inhibited the growth of duckweed [12], but CuO NPs at 125 mg/kg exhibited no impact on the yield of bell pepper [13]. The application routes can also determine the impact of CuO NPs on crop growth. Foliar application of 40 µg/mL CuO NPs inhibited the germination rate of lettuce and subsequent plant growth [14], while no adverse effect was found in the root application route [15]. Few studies have explored the differential regulation of plant growth by CuO NPs as a function of particle size. For example, as compared to granular CuO, bulk CuO caused cell membrane damage to corn seedlings [16].

Soybean is an important crop and widely cultivated [17], and it also plays a crucial role in the human food supply by providing rich proteins [18]. Additionally, it contributes approximately 40% of the annual oilseed crop yield worldwide [19]. In terms of economics, soybeans’ contribution to a variety of industries is worth USD 30 billion worldwide [20]. Hence, enhancing soybean growth and nitrogen fixation is considered one of the most effective ways to increase yield and global nitrogen cycling.

Nitrogen (N) is an essential nutrient element for plant growth and development [21], and N deficiency can limit agriculture production on a global scale [22]. N metabolism is one of the basic physiological processes determining crop growth, quality, and yield [23], and N deficiency is a limiting factor that can inhibit plant growth [24]. Although N_2_ in the atmosphere is sufficient, it must be converted into ammonia (NH_4_^+^) or nitrate (NO_3_^−^) for plant utilization [25]. NH_4_^+^ and NO_3_^−^, as signaling molecules, participate in a series of physiological metabolism processes and play an essential role in plant growth and development [26]. Most chemical forms of nitrogen can be converted to NO_3_^−^ rapidly in aerobic soil, and NO_3_^−^ is also the main form that plants can take up from soil [27]. Once accumulating in root systems, NO_3_^−^ can be assimilated into NH_4_^+^ and organic matters with the participation of nitrate reductase (NR) and nitrite reductase (NiR), and then the NH_4_^+^ is converted into nitrogenous organics by the GS/GOGAT cycle [28]. These enzymes (NR, NiR, GS, GOGAT) are critical and involved in N metabolism, and their activities determine the N accumulation in plants. Zhang et al. reported that nano-MoO_3_ increased the activities of essential enzymes associated with nitrogen metabolism and nitrate utilization in rice [27].

NPs regulating soybean growth and nitrogen assimilation have been reported previously. Zhou et al. found that NiO NPs at 50 mg/kg increased the activities of nitrate reductase, urease, glutamine synthetase, and glutamate synthesis in plants [29]. The use of ZnO NPs at 25 mg/kg increased the biomass and nitrogen assimilation of *Phaseolus vulgaris* [30]. Saheli et al. reported that Mn NPs acted as cofactors in a series of enzymatic reactions in the process of assimilating nitrate into organic nitrogen compounds and subsequently improved the nitrogen content in plants [31]. MoS_2_ NPs released molybdenum and sulfur, both of which participate in enzymes involved in nitrogen metabolism, and then altered soybean growth [32]. Only a few studies have focused on the relationship between CuO NPs and soybeans. For example, Suman et al. reported that the toxicity of CuO NPs to soybean seedlings was dependent on size and use doses [33]. Hence, although a large body of research has been conducted on NPs, there is still a lack of understanding of the differential regulation of soybean growth and nitrogen assimilation by different sizes of CuO NPs.

The objective of the present work was to investigate the physiological responses of soybeans upon exposure to different sizes of CuO NPs. Particularly, the pattern of N_2_ assimilation in soybeans as affected by different sizes of CuO NPs was elucidated. At harvest, physiological parameters, including biomass, nitrogen assimilation-related enzymic activities (e.g., NR, NiR, GS, GOGAT), and nitrogenous compounds (e.g., NO_3_^−^-N, NH_4_^+^-N, GSH, amino acids, proteins), were measured across all the treatments. Additionally, the contents of both micro- and macro-nutrients in soybean shoots and roots as a function of particle size were determined. Overall, the present study contributes to a better understanding of the potential implications of different sizes of CuO NPs in altering nitrogen assimilation in legume crops.

## 2. Materials and Methods

### 2.1. Nanoparticle Characterization

CuO NPs (20 nm and 50 nm) used in this study were purchased from Brofos Nanotechnology (Ningbo) Co., Ltd. (Ningbo, China). The purity was ≥99.9%, and the specific surface area was 27.67 m^2^/g. Scanning electron microscopy (SEM) images show that two sizes of CuO NPs were irregular (Figure S1). The average particle size was 25 ± 4.5 for 20 nm CuO NPs and 47 ± 12.8 for 50 nm CuO NPs (Figure S1).

### 2.2. Pot Experiment

#### 2.2.1. Concentration Optimization of CuO NPs

A pre-experiment was set up with different concentrations of two sizes of (20 and 50 nm) CuO NPs to determine the optimal application dose that positively affects the physiological responses of soybeans (*G. max* (L.) Merrill). Soybean seeds were germinated and grown in the soil amended with different doses (1, 5, 10, 25, and 50 mg/kg) of CuO NPs under greenhouse conditions (14/10 h light/dark cycle, 26/20 °C light/dark cycle, humidity was 50~60%). Each pot received 50 mL of tap water daily for 21 days. At harvest, the fresh/dry biomass (Tables S1 and S2) and nitrate nitrogen contents (Table S3) of soybeans were used to determine that 1 and 10 mg/kg doses were the optimal concentrations of different sizes of CuO NPs for enhancing soybean growth.

#### 2.2.2. Pot Experiment with Optimal Concentrations of CuO NPs

The pot experiment was repeated with the optimal concentration of CuO NPs under the same growth conditions. Five biological replicates were set in each treatment. After 21 days, the soybean seedlings were removed from the soil and thoroughly washed with tap water, followed by two-time distilled water washes. Phenotypic images and basic physiological parameters (weight and length) were recorded across all the treatments. Then, all the fresh samples were stored at −80 °C until further biochemical analysis. The whole experiment was performed twice to make the results repeatable.

### 2.3. Enzyme Activity Related to Nitrogen Assimilation

The activities of nitrate reductase (NR; consumption of 1 μmol NADH per gram of fresh tissue per hour was defined as one unit of NR enzyme activity) and glutamine synthetase (GS; a change of 0.01 in absorbance at 540 nm per gram of tissue per minute was defined as a unit of GS enzyme activity) were measured in shoots and roots across all the treatments by following the manufacturer’s instructions (Beijing Boxbio Science & Technology Co., Ltd., Beijing, China).

#### 2.3.1. Nitrite Reductase

Nitrite reductase (NiR) activity was measured as described in Miflin et al. [34]. Briefly, 0.1 g of fresh tissues were homogenized in 1 mL of phosphate buffer (PB), and then the mixture was centrifuged at 8000 rpm for 10 min at 4 °C. The reaction buffer containing 0.12 mL 0.1 mol/L NaCl, 1 mL 0.1 mol/L PB (pH = 6.5), 0.1 mL 0.1 mol/L NaNO_2_, 0.06 mL methyl viologen (MV), and 0.32 mL 0.1 mol/L Na_2_S_2_O_4_ was mixed with 0.4 mL of supernatant and then incubated at 50 °C for 30 min. Afterwards, the reaction mixture was vigorously shaken until the color of the MV disappeared. The absorbance of the residual NO_2_^−^ in the reaction mixture was measured using a UV-Vis spectrophotometer (GENESYS 180, Thermo Fisher Scientific, Waltham, MA, USA). The amount of enzyme required to reduce 1 μmol NO_3_^−^ per gram of fresh tissue per hour was defined as one unit of NiR enzyme activity.

#### 2.3.2. Glutamate Synthase

Glutamate synthase (GOGAT) activity was measured as described in Wang et al. [35], with some minor modifications. Briefly, 0.5 g shoot and 0.1 g root tissues were homogenized in 1.5 mL 0.1 mol/L Tris-HCl buffer (pH = 8.0) and centrifuged at 10,000 rpm for 20 min. Afterwards, 0.3 mL of supernatant was added to a reaction buffer containing 0.4 mL 20 mmol/L L-glutamine, 0.5 mL 20 mmol/L α-ketoglutarate, 0.1 mL 10 mmol/L KCl, 0.2 mL 3 mmol/L NADH, and 1.5 mL 25 mmol/L Tris-HCl buffer (adding the reaction system to 3 mL). The absorbance was measured at a wavelength of 340 nm every 20 s for 300 s. The consumption of 1 μmol NADH per gram of tissue per minute was defined as a unit of GOGAT enzyme activity.

### 2.4. Determination of the Content of Nitrogenous Compounds

#### 2.4.1. Nitrate-N

The Nitrate-N (NO_3_^−^-N) concentration was determined using the salicylic acid method as described in Cataldo et al. [36], with some minor modifications. Briefly, 0.1 g of dry tissues were homogenized in 1 mL of DI water and incubated at 45 °C for 1 h, then the mixtures were centrifuged at 10,000 rpm for 25 min at room temperature. Afterwards, 0.2 mL of supernatant was gently mixed with 0.8 mL of 5% (*w*/*v*) salicylic acid in concentrated H_2_SO_4_. After 30 min, 19 mL 2 mol/L NaOH was added slowly. Then the absorbance was measured at a wavelength of 340 nm. The Nitrate-N concentration was determined from a standard curve and expressed as mg NO_3_^−^-N per g dry weight.

#### 2.4.2. Ammonia-N

The ammonia-N (NH_4_^+^-N) was assayed by the phenol–hypochlorite method as described in Weatherburn [37] after plant tissue digestion in sulfuric acid and H_2_O_2_. Briefly, 1 mL of digest was added into 1 mL of EDTA-methyl red, and 0.3 mol/L NaOH was added gently to make the solution turn yellow, then 5 mL of phenol plus nitroprusside and 5 mL of alkaline sodium hypochlorite were added sequentially. Afterwards, DI water was used to make the final reaction volume 50 mL. After 1 h, the absorbance was measured at a wavelength of 625 nm.

#### 2.4.3. Amino Acid and Protein

Amino acid and soluble protein content were measured by following Xu et al. [38]. Briefly, 1 g of shoot and root tissues were mixed with 5 mL 10% acetic acid, and then the mixture was diluted to 100 mL using DI water. After filtration, 4 mL of extracts were mixed with 1 mL 20 g/L ninhydrin and 1 mL of PBS (pH = 8.0) and incubated at 90 °C in a water bath for 40 min. The absorbance was measured at a wavelength of 350 nm to determine the amino acid content. For the protein content, 1 mL of extract was mixed with 5 mL of Coomassie brilliant blue reagent for 15 min. The absorbance was measured at a wavelength of 595 nm to determine the soluble protein content.

### 2.5. Soil Enzyme Activity

#### 2.5.1. Soil Urease

Soil urease (S-UE) enzyme activity was determined by the colorimetric method of sodium phenolate–sodium hypochlorite as described by Li et al. [39]. Briefly, 5 g of air-dried soil was mixed with 1 mL of toluene, then mixed with 10 mL of 100 g/L urea and 20 mL of citrate buffer (pH = 6.7) for 15 min, then incubated at 37 °C for 24 h. Afterwards, 4 mL 1.35 mol/L sodium phenolate and 3 mL of sodium hypochlorite (available chlorine was 0.9%) were added to the mixture for 20 min, and then DI water was added to make the final volume 50 mL. The absorbance was measured at a wavelength of 578 nm. The activity of S-UE was expressed as ammonia (mg) produced per gram of air-dried soil hydrolysis per day.

#### 2.5.2. Soil Neutral Phosphatase

Soil neutral phosphatase (S-NP) enzyme activity was determined by the disodium phenyl phosphate colorimetric method as described by Li et al. [39]. Briefly, 2 g of air-dried soil was mixed with 1 mL of C_7_H_8_ and then shaken for 15 min. Afterwards, the mixture was mixed with 5 mL of C_6_H_5_Na_2_PO_4_ and incubated at 37 °C for 24 h. Four mL of filtrate was added to 5 mL of citrate buffer (pH = 7.0) and 200 μL 12.5 g/L C_6_H_2_Br_2_CINO, then diluted to 50 mL with DI water. The reaction was left on the bench for 30 min before the measurement. The absorbance was measured at a wavelength of 660 nm. The activity of S-NP was expressed as phenols (mg) produced per gram of air-dried soil hydrolysis per day.

#### 2.5.3. Soil Polyphenol Oxidase

Soil polyphenol oxidase (S-PPO) enzyme activity was determined by the colorimetric method as described by Li et al. [39]. Briefly, 1 g of air-dried soil was mixed with 10 mL of 1% pyrogallol and incubated at 30 °C for 24 h, then 4 mL of citrate–phosphate buffer (pH = 4.5) and 35 mL of ethyl ether were added and extracted for 30 min. The absorbance was measured at a wavelength of 430 nm. The activity of S-PPO was expressed as pyrogallol (mg) produced per gram of air-dried soil per day.

#### 2.5.4. Soil Sucrase

Soil sucrase (S-SC) enzyme activity was determined by 3,5-Dinitrosalicylic acid colorimetry, as described by Li et al. [39]. Briefly, 5 g of air-dried soil were mixed with 15 mL 80 g/L C_12_H_22_O_11_, 5 mL of PB (pH = 5.5), and 200 μL of C_7_H_8_, and then the mixture was incubated at 37 °C for 24 h. Afterwards, 1 mL of filtrate was mixed with 3 mL of 3,5-Dinitrosalicylic acid (DNS) reagent and incubated in a boiled water bath for 5 min, then in an ice bath for 10 min. The reaction solution was diluted to 50 mL with DI water before being measured at a wavelength of 540 nm. The activity of S-SC was expressed as glucose (mg) produced per gram of air-dried soil hydrolysis per day.

### 2.6. Element Content Measurement

The element contents in soybean tissues and soil samples across all the treatments were measured by following Xu et al. [38]. Plant dry tissues or air-dried soil samples were digested in a mixture of HNO_3_ and H_2_O_2_ for the measurement of micro- and macro-nutrients. Briefly, approximately 0.1 g of dry shoots, roots, or soil were mixed with 5 mL of concentrated nitric acid and heated at 115 °C for 90 min. Then, the digests were cooled for 20 min before adding 4 mL of 30% (*v*/*v*) H_2_O_2_. Afterwards, the digests were heated at 115 °C until they were concentrated to approximately 1 mL, which was diluted to 50 mL with DI water before the measurement. Finally, the concentration of elements (K, Mn, Fe, P, Cu, etc.) was measured by inductively coupled plasma optical emission spectrometry (ICP-OES, iCAP 7000, Thermo Fisher, USA). The elemental contents in plant tissues or soil were expressed in mg/kg of dry weight.

### 2.7. Statistical Analysis

All of the values represent the mean ± standard deviation. Different letters within each NP concentration indicate significant differences at *p* ≤ 0.05 according to one-way analysis of variance (ANOVA), followed by the least significant difference (LSD) test. Letters are only reported when differences among means are statistically significant. Data analysis was performed by IBM SPSS Statistics 26.

## 3. Results and Discussion

### 3.1. Effects of CuO NPs on Soybean Biomass

**Figure 1:**
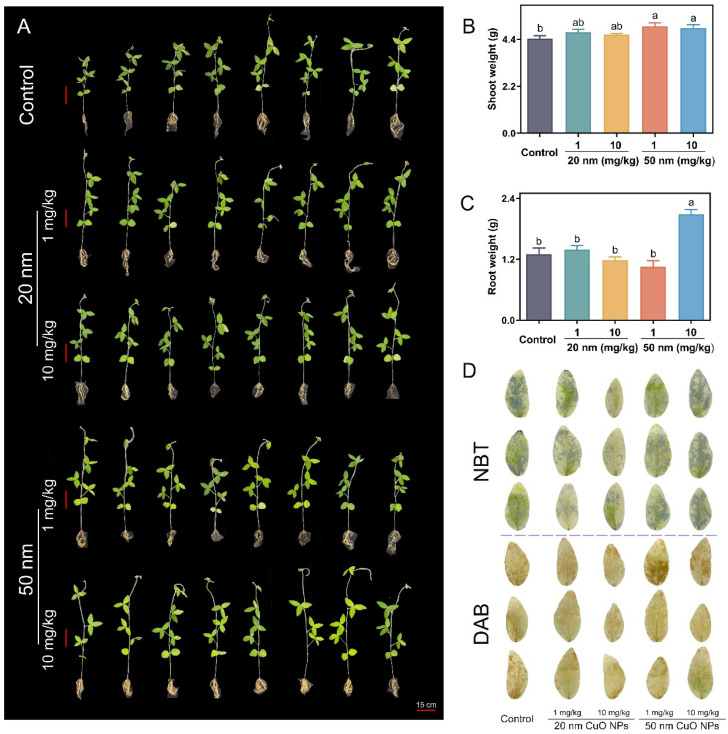
Phenotypic images (**A**), fresh shoot weight (**B**), root weight (**C**), and NBT staining and DAB staining (**D**) of soybean plants treated with 20 and 50 nm CuO NPs at 1 and 10 mg/kg for three weeks. Values are means of 8 plants ± SD, and different letters in each panel represent significantly different values at *p* < 0.05. Scale = 15 cm.

Both sizes of CuO NPs increased the soybean biomass regardless of the use doses (Table S4), and phenotypic images show that the CuO NP treatments promoted soybean growth better (Figure 1A). In particular, in the 1 mg/kg NP treatments, 20 and 50 nm CuO NPs led to 6.69% and 12.83% increases in the fresh weight of shoots (Figure 1B), respectively, relative to the control. Similar results were also evident in the 10 mg/kg NP treatments, where 20 and 50 nm CuO NPs increased the shoot fresh weight by 4.4% and 10.97%, respectively (Figure 1B). It is interesting to find that root application of different sizes of CuO NPs affected the shoot biomass more than the root across all the treatments. For the root, only 50 nm CuO NPs at 10 mg/kg positively increased the root fresh weight by 60.56% as compared to the control, while the other three treatments had no impact (Figure 1C). Biomass is an essential parameter to evaluate the efficiency of promoting the effects of metal-based NPs on crop growth [30]. In order to prove the positive growth effect of NPs on soybeans, reactive oxygen species (ROS) staining was performed on leaves and roots. The images of ROS staining demonstrated that CuO NPs enhanced the soybean growth without causing any oxidative stresses (Figure 1D and Figures S2–S4). Moreover, GSH is one of the antioxidants in plants that can remove excess ROS under abiotic stresses. The GSH content was only slightly increased in shoot and root tissues upon NP applications (Figure S5). Guan et al. reported that root application of CuO NPs increased the wheat biomass via improving the photosynthetic efficiency [25]. Abdel et al. also found that TiO_2_ NPs at 0.01% (*w*/*w*) significantly increased the shoot height, leaf area, and root dry weight of broad bean plants [40].

### 3.2. Effects of CuO NPs on Nitrate Reduction in Soybeans

As the main nitrogen source for plant uptake and utilization, nitrate nitrogen (NO_3_^−^-N) is not only a nutrient but also a signaling molecule, which plays a critical regulatory role in breaking seed dormancy and leaf growth [25]. The NO_3_^−^-N in the soil is taken up by the root cells via a specific nitrate transporter, where it is eventually reduced to ammonia nitrogen under the action of NR and NiR.

**Figure 2:**
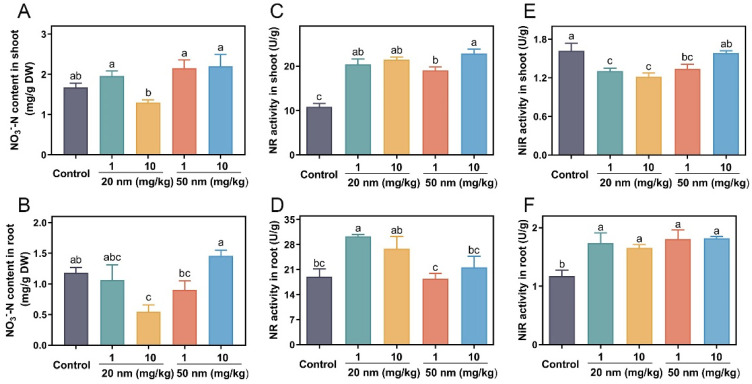
NO_3_^−^ content (**A**,**B**), NR enzyme activity (**C**,**D**), and NiR enzyme activity (**E**,**F**) in soybean shoots and roots in the 20 and 50 nm CuO NP treatments at 1 and 10 mg/kg for three weeks. Error bars represent the standard error of 5 replicates, and the different letters in each panel represent significantly different values at *p* < 0.05.

In comparison with the control, both 20 nm and 50 nm CuO NPs at 1 mg/kg increased the content of NO_3_^−^-N in shoots by 17.1% and 28.5% (Figure 2A), respectively. At 10 mg/kg, the addition of 50 nm CuO NPs increased the NO_3_^−^-N content in shoots by 31.4%, while 20 nm CuO NPs significantly reduced the content of NO_3_^−^-N in shoots (Figure 2A). In roots, the addition of 20 nm CuO NPs decreased the NO_3_^−^-N content with increasing the use doses, while 50 nm CuO NPs significantly increased the NO_3_^−^-N content with increasing the use doses (Figure 2B); particularly, root application of 50 nm CuO NPs at 10 mg/kg increased the root NO_3_^−^-N content by 23.5% relative to the control (Figure 2B). The possible explanations that both sizes of CuO NPs altered the NO_3_^−^-N content in roots can be ascribed to either the increases in the activities of NR and NiR enzymes or the timing to activate the NR and NiR enzymes not being coordinated with that of NO_3_^−^-N production.

NR is the first enzyme involved in N assimilation and is also considered a limiting factor for plant growth. In shoots, the NR enzyme activity was increased by 88.2% and 75.6% in the 20 nm and 50 nm CuO NP treatments at 1 mg/kg and increased by 98% and 110.9% at 10 mg/kg, respectively, relative to the control (Figure 2C). In roots, only 20 nm CuO NP treatments with 1 and 10 mg/kg increased the activity of the roots’ NR enzyme by 59.7% and 41.5%, respectively, relative to the control, while 50 nm CuO NP treatments had no impacts regardless of the use doses (Figure 2D). The increase in NR enzyme activity enhanced the NO_3_^−^-N to NO_2_^−^-N conversion in shoots and roots as a function of CuO NPs. The 20 nm CuO NP treatments increased the NR enzyme activities more than the 50 nm NP treatments in both shoots and roots, which could be the reason for the decreases in the NO_3_^−^-N content in 20 nm CuO NP-treated tissues. The NiR enzyme reduces the nitrate reduction product of the previous step from NO_2_^−^-N to NH_4_^+^-N [41]. All the CuO NP treatments decreased the NiR enzyme activities in shoots (Figure 2E); however, the NiR enzyme activity in roots was increased by 41~48% and 54~55% as affected by 20 and 50 nm CuO NPs, respectively, relative to the control.

In general, 50 nm CuO NPs accumulated more NO_3_^−^-N in soybean tissues than 20 nm NPs, and the increase in NO_3_^−^-N contents suggests that under the catalysis of NR and NiR enzymes, more NO_3_^−^-N was reduced to NO_2_^−^-N, NH_4_^+^-N, and other nitrogen compounds, all of which could boost the nitrogen assimilation pathway in soybeans.

### 3.3. Effects of CuO NPs on Ammonia Assimilation in Soybeans

**Figure 3:**
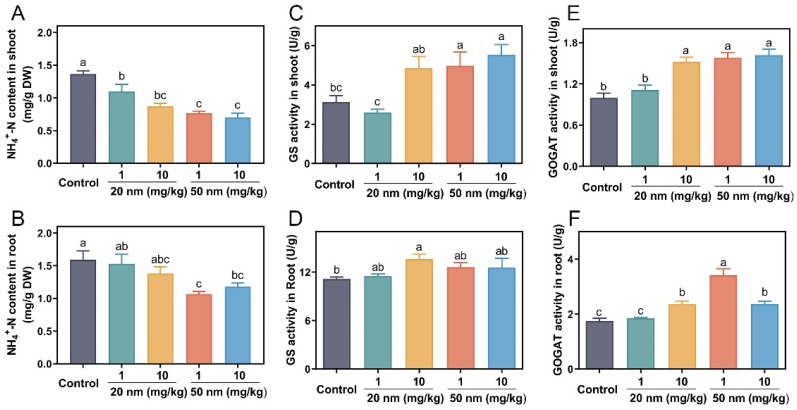
NH_4_^+^ content (**A**,**B**), GS enzyme activity (**C**,**D**), and GOGAT enzyme activity (**E**,**F**) in soybean shoots and roots in the 20 and 50 nm CuO NP treatments at 1 and 10 mg/kg for three weeks. Error bars represent the standard error of 5 replicates, and the different letters in each panel represent significantly different values at *p* < 0.05.

The presence of CuO NPs lowered the NH_4_^+^-N contents in both shoots and roots when compared to the control; additionally, the lowest NH_4_^+^-N contents were evident in the 50 nm CuO NP treatments (Figure 3A,B). At 1 mg/kg, the addition of 20 nm CuO NPs decreased the NH_4_^+^-N contents by 19.6% and 3.7% in shoots and roots, respectively, relative to the control, while 50 nm CuO NPs decreased the NH_4_^+^-N contents by 43.9% and 32.8% in shoots and roots, respectively, relative to the control. At 10 mg/kg, 20 and 50 nm CuO NPs decreased the NH_4_^+^-N contents by 36.1~48.4% and 13~25.9% in shoots and roots, respectively, relative to the control (Figure 3A,B). It is reasonable to speculate that the decreases in the NH_4_^+^-N contents were because the CuO NP treatments promoted ammonia assimilation and accelerated the reduction of NO_3_^−^-N in plants. Thus, the key enzymes (GS and GOGAT) in ammonia assimilation were determined across all the CuO NP treatments.

At 1 mg/kg, both 20 and 50 nm CuO NPs increased the GS enzyme activity in both shoots and roots, except for the 20 nm CuO NP treatment, which decreased the shoot GS activity by 16.8% relative to the control (Figure 3C), while the 50 nm CuO NP treatment increased the GS activity by 59.4% and 13.3% in shoots and roots, respectively. At 10 mg/kg, the highest GS activity in shoots was evident in the 50 nm CuO NP treatments, where the GS activity was 76.9% higher than the control (Figure 3C); the 20 nm CuO NP treatments increased the GS activity by 22.3% (Figure 3D) in roots. Changes in the GOGAT enzyme activity in roots were similar to the GS enzyme activity as a function of CuO NPs. In shoots, root application of 10 mg/kg 20 nm CuO NPs increased the GOGAT enzyme activities by 53.3%, while 50 nm CuO NPs increased them by 58.6% and 63% at 1 and 10 mg/kg, respectively, relative to the control (Figure 3E). In roots, at 1 mg/kg, 50 nm CuO NP treatments increased the activity of the GOGAT enzyme by 95%, while 20 nm CuO NP treatments had no impact. At 10 mg/kg, the activity of the GOGAT enzyme was increased by 35% by both sizes of CuO NPs (Figure 3F).

Ammonia nitrogen (NH_4_^+^-N) is toxic and cannot be accumulated in plants; it needs to be assimilated to transform into a form that can be absorbed and utilized by plants [42]. The assimilation of NH_4_^+^-N in plants mainly depends on the GS/GOGAT cycle, which converts the inorganic N into organic N compounds [43]. The content of NH_4_^+^-N in roots was higher than in shoots, demonstrating that the activities of GS and GOGAT enzymes in roots were usually significantly higher than those in shoots.

### 3.4. Amino Acid and Soluble Protein Content

**Figure 4:**
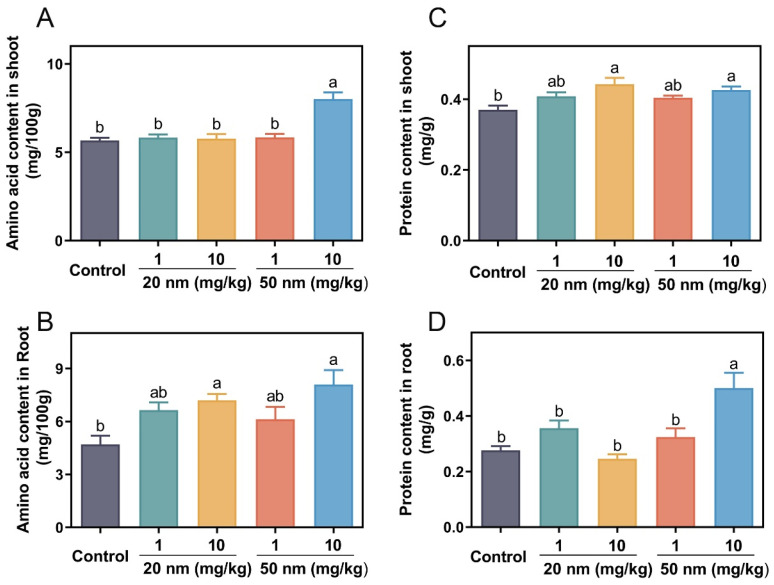
Amino acid content (**A**,**B**) and protein content (**C**,**D**) in soybean shoots and roots in the 20 and 50 nm CuO NP treatments at 1 and 10 mg/kg for three weeks. Error bars represent the standard error of 5 replicates, and the different letters in each panel represent significantly different values at *p* < 0.05.

Proteins and amino acids are the final products of plant N metabolism, and their content can preliminarily evaluate the absorption and utilization of N by plants. In shoots, only 50 nm CuO NPs at 10 mg/kg increased the amino acid contents by 41.4%, while other treatments had no impact (Figure 4A). In roots, both sizes of CuO NP treatments increased amino acid contents significantly. At 1 mg/kg, 20 and 50 nm CuO NP treatments increased the amino acid content by 41% and 30%, while increasing it by 53% and 72.1% at 10 mg/kg, respectively, relative to the control (Figure 4B). Amino acids, as the basic units of protein, play a critical role in plant metabolic processes [38]. It is demonstrated that TiO_2_ NPs increase the amino acid contents and enzymatic antioxidant activities in salt-stressed plants [40]. Thus, the addition of CuO NPs within appropriate use doses could maintain the healthy status of legume crops via elevating amino acid levels. Similarly, CuO NP treatments also increased the protein contents in shoots. For instance, 20 nm CuO NP treatments increased the protein contents by 10.3% and 19.5% at 1 and 10 mg/kg, while 50 nm CuO NP treatments increased them by 9.2% and 15.1%, respectively, relative to the control (Figure 4C). In roots, only 50 nm CuO NP treatments increased protein contents by 81.2% relative to the control, while others had no impact (Figure 4D).

### 3.5. Soil Enzyme Activity

**Figure 5:**
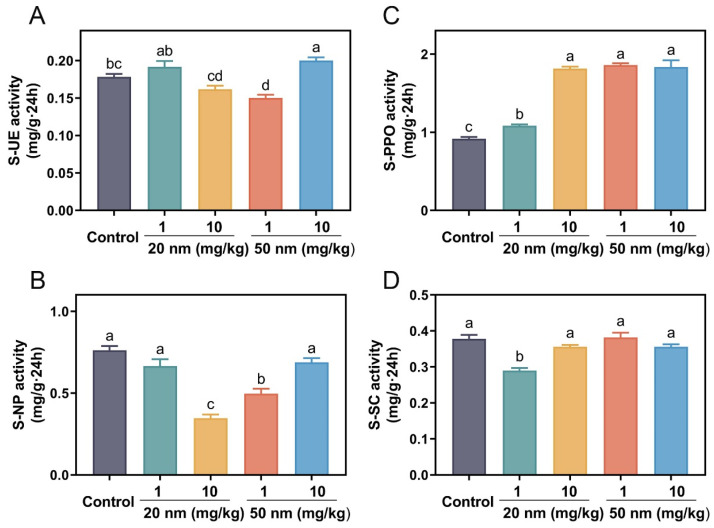
Soil urease (S-UE, **A**), soil neutral phosphatase (S-NP, **B**), soil polyphenol oxidase (S-PPO, **C**), and soil sucrase (S-SC, **D**) enzyme activity in the 20 and 50 nm CuO NP treatments at 1 and 10 mg/kg for three weeks. Error bars represent the standard error of 5 replicates, and the different letters in each panel represent significantly different values at *p* < 0.05.

For the S-UE activity, no significant impact was found across all the CuO NP treatments in terms of use doses and particle sizes, except the 50 nm CuO NP treatments, where the S-UE activity was significantly reduced by 15.5% at 1 mg/kg and increased by 10.9% at 10 mg/kg, as compared to the control (Figure 5A). Soil urease can catalyze the hydrolysis of urea into NH_3_ and CO_2_ in the soil [44]; thus, the activity of S-UE determines the amount of NH_3_ that can be taken up and utilized by plants [45]. The decreases in the S-UE activity in the CuO NP treatments might be the reason that the NO_3_^−^-N contents were reduced in roots. The pattern of the changes in the S-NP activities as a function of CuO NPs was similar to the NO_3_^−^-N content in roots (Figure 2B). At 10 mg/kg, the 20 nm CuO NP treatments reduced the S-NP activity by 54.4%, while the 50 nm CuO NP treatment with 1 mg/kg reduced the S-NP activity by 35% relative to the control (Figure 5B). S-NP activity was positively correlated with soil N content and can affect the availability of P in the soil directly [46].

At both use doses, the 20 nm CuO NP treatments increased the S-PPO activity by 18.2% and 98%, while the 50 nm CuO NP treatments increased its activity by 103% and 100%, respectively, relative to the control (Figure 5C). Excess amounts of NO_3_^−^ and NH_4_^+^ in the soil could inhibit the activities of fungi, which secrete polyphenol oxidase [47]; thus, the increased S-PPO activities might be caused by the increased uptake of NO_3_^−^ and NH_4_^+^ by roots as a function of CuO NPs. The increase in S-PPO activities could provide more energy for soil microorganisms, which was conducive to the increase of microbial biomass carbon [48]. For the S-SC activity, no significant impact was found across all the CuO NP treatments in terms of use doses and particle sizes, except the 20 nm CuO NP treatments, where the S-SC activity was significantly reduced by 23% relative to the control (Figure 5D).

### 3.6. Cu Uptake and Accumulation in Soybeans

**Figure 6:**
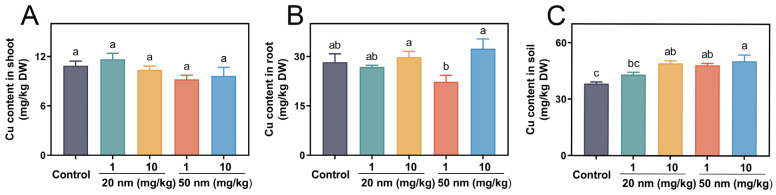
Cu content in shoots (**A**), roots (**B**), and soil samples (**C**) in the 20 and 50 nm CuO NP treatments at 1 and 10 mg/kg for three weeks. Error bars represent the standard error of 5 replicates, and the different letters in each panel represent significantly different values at *p* < 0.05.

The Cu contents in shoots, roots, and soil samples in the control were 10.89 ± 1.24, 8.29 ± 5.76, and 38.32 ± 1.96 mg/kg dry weight, respectively (Figure 6). In shoots, CuO NP treatments reduced the Cu contents slightly but not significantly (Figure 6A). In roots, at 1 mg/kg, the 50 nm CuO NP treatment decreased the Cu contents by 21.1%, while the 20 and 50 nm CuO NP treatments at 10 mg/kg increased the Cu contents by 5.5% and 14.5%, respectively, relative to the control (Figure 6B). Liu et al. reported that CuO NP (40 nm) suspensions at 2 mg/L increased the Cu contents in roots [49]. In addition, Wang et al. also reported that CuO NPs (40 nm) were observed in the meristematic cells of the root zone and increased the Cu contents by 32.3-fold in dry roots as compared to the control [50]. In the soil, the addition of CuO NPs increased the Cu contents in a dose-dependent fashion after harvesting. At 1 mg/kg, the 20 and 50 nm CuO NP treatments increased the Cu contents by 12.6% and 28.6%, while increasing them by 28.2% and 31.4% at 10 mg/kg, respectively, relative to the control (Figure 6C), suggesting that at the same use dose, the 50 nm CuO NP treatments resulted in more Cu accumulation in the soil than the 20 nm CuO NP treatment. Other nutrient contents in soybean shoots, roots, and soil samples across all the CuO NP treatments are provided in Table S5.

A previous study indicated that the NP size can determine the uptake and utilization of elements by plants and that smaller nanoparticles (≤20 nm) are more likely to be taken up and accumulated by plants [51]. Thus, higher Cu contents in shoots were observed in the 20 nm CuO NP treatments. However, the larger NP size might induce the formation of large pores in root cell walls [52,53] and subsequently enter the plant tissues via the wounds, which could explain the reasons that the 50 nm CuO NP treatments increased the Cu contents in roots more than the 20 nm treatments.

## 4. Conclusions

**Figure 7:**
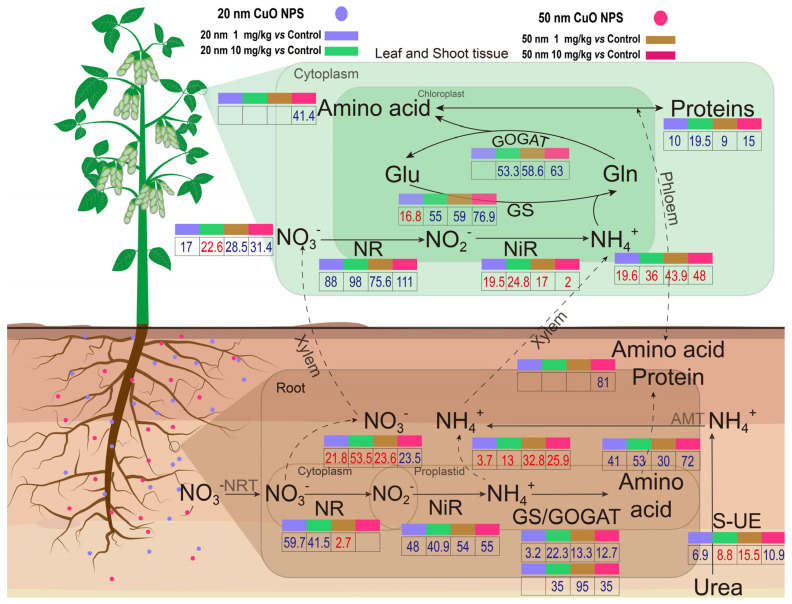
A schematic diagram of the size effects of CuO NPs on nitrogen fixation in a soybean plant. The numbers in the figure show the percentage change of NP treatments relative to the control (numbers with blue color indicate an increase; red color indicates a decrease).

The results showed that the use of 20 and 50 nm CuO NP treatments at low doses could increase the growth and nitrogen assimilation of soybeans in terms of N-associated enzyme activities and nitrogenous compounds (Figure 7). Moreover, the use doses of CuO NPs did not significantly alter the Cu contents in soybean tissues, although a dose-dependent fashion of Cu contents in soil samples was evident. More importantly, our findings demonstrated that 20 and 50 nm CuO NPs were beneficial to the early growth and increased the nitrogen assimilation abilities of soybeans at low doses without causing any oxidative stresses. At the same use dose, the 50 nm CuO NP treatments outperformed the 20 nm CuO NP treatments, as determined by crop growth and nitrogen assimilation. Overall, nanoscale Cu-based agrochemicals have exhibited great potential for application in sustainable agriculture, particularly in legume crops in this case, and could also potentially reduce the risk of excessive use of nitrogen fertilizer in the agricultural environment.

## Data Availability

Data are contained within the article and Supplementary Materials.

## Supplementary Materials

The following supporting information can be downloaded at https://www.mdpi.com/article/10.3390/nano14090746/s1, Detailed information, including three experiments, four descriptions, five figures, and five tables, is provided in the Supplementary Materials file. Figure S1: Scanning electron microscopy (SEM) scan of different sizes of CuO NPs in deionized water; Figure S2: DCFH-DA staining of soybean blades under different sizes of CuO NPs at 1 and 10 mg/kg; Figure S3: DAB and NBT staining of root tips under different sizes of CuO NPs at 1 and 10 mg/kg; Figure S4: DCFH-DA staining of soybean root under different sizes of CuO NPs at 1 and 10 mg/kg; Figure S5: GSH content in soybean shoot and root under different sizes of CuO NPs at 1 and 10 mg/kg; Table S1: length and biomass of soybean plants under the treatment of 20 nm CuO NPs in pre-experiment; Table S2: length and biomass of soybean plants under the treatment of 50 nm CuO NPs in pre-experiment: Table S3: NO_3_^−^-N content in shoot and root of soybean plants in pre-experiment; Table S4: length of soybean plants; Table S5: Element content in soybean shoot, root and soil. (See Refs. [50,54,55]).
