# Supplementary material for: Size Effects of Copper Oxide Nanoparticles on Boosting Soybean Growth via Differentially Modulating Nitrogen Assimilation
Source: Nanomaterials (Basel). 2024 Apr 25;14(9):746. doi: 10.3390/nano14090746 (PMC11085672; doi:10.3390/nano14090746)
Supplement: Supplementary file 1 [file nanomaterials-14-00746-s001.zip › nanomaterials-2959088-supplementary.pdf]

Supporting information

for

Size Effects of Copper Oxide Nanoparticles on Boosting Soybean Growth via Differentially Modulating Nitrogen Assimilation

Yaozu Guo[1], Hao Li[1], Yi Hao[1], Heping Shang[1], Weili Jia[2, *], Anqi Liang[1], Xinxin Xu[1], Chunyang Li[1], Chuanxin Ma[1, *]

[1]Key Laboratory for City Cluster Environmental Safety and Green Development of the Ministry of Education, Institute of Environmental and Ecological Engineering, Guangdong University of Technology, Guangzhou 510006, China; chuanxin.ma@gdut.edu.cn

[2]SCNU Environmental Research Institute, School of Environment, Guangdong Provincial Key Laboratory of Chemical Pollution and Environmental Safety & MOE Key Laboratory of Theoretical Chemistry of Environment, South China Normal University, Guangzhou 510006, China; weili.jia@m.scnu.edu.cn

Number of pages: 13

Number of figures 5

Number of tables: 5

Section S1. Soil condition

The soil used was collected the top 20 cm of the soil from Haizhu wetland, Guangzhou, Guangdong province, China, which was used as a control treatment without any fertilizer or pesticide input. Fresh soil was air-dried at room temperature for 5 days and then passed through a 2 mm sieve. The Cu content was 38.32 ± 1.96 mg/kg.

Section S2. DAB and NBT staining of blades

The hydrogen peroxide (H_2O_2) of blades were assay using 3',3', -diaminobenzidine (DAB) following Shaw et al[1] (nano-CuO stress induced modulation of antioxidative)., with minor modification. Briefly, the cotyledons from the same position were selected, infiltration in 5mM DAB staining solution for one night, chlorophylls were removed by infiltration with ethanol, then images were captured after boiling in water bath for 10 min.

The roots were stained for 5 h with 0.1% NBT in 10 mM potassium phosphate (pH=7.0) as described by Nguyen et al[2], then decolorized by 95% alcohol in 80 °C water bath.

Section S3. DCFH-DA staining

The method of ROS staining was following wang et al.[3], with minor modifications. Briefly, 5 roots tips and blades from each treatment group were randomly placed in tubes which 10 μmol/L 7′-dichlorodihydrofluorescein–diacetate (DCFH-DA) solution. And washed 3 times at least by phosphate buffered saline (PBS) after 15 min. Then the tissues were imaged using a Spinning Disc Confocal microscope with excitation wavelength at 488 nm and emission wavelength at 522 nm with settings of 200 ms. All process was performed in the darkroom.

Section S4. GSH content

The GSH contents were according to manufacturer's instructions (Beijing Boxbio Science & Technology Co. Ltd.).

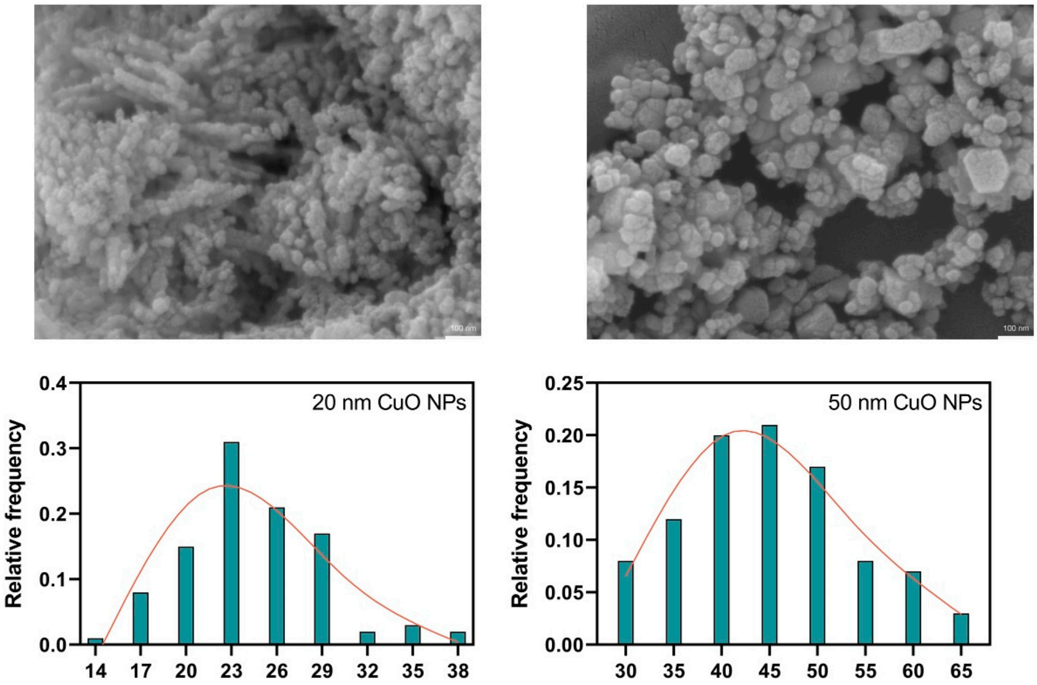

Figure S1 Scanning electron microscopy (SEM) scan of CuO NPs (left was 20 nm, right was 50 nm) in deionized water.

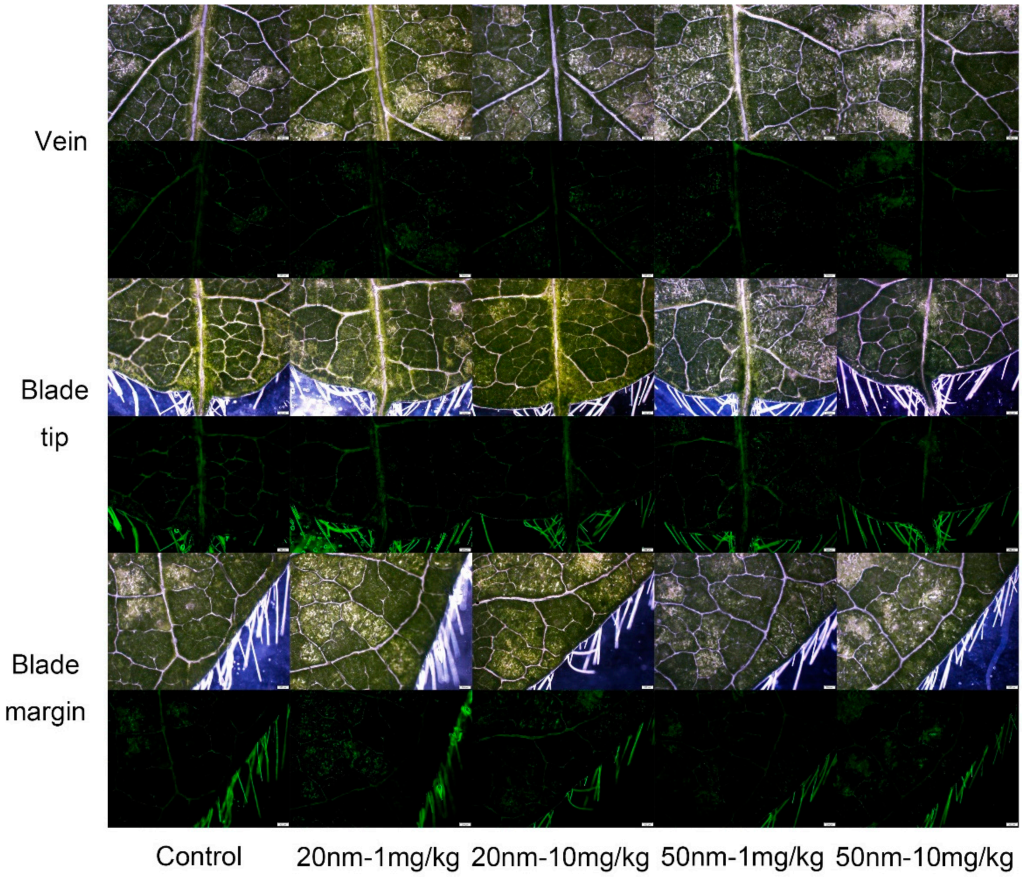

Figure S2 DCFH-DA staining of soybean blades under the treatments of 1 and 10 mg/kg of 20 and 50 nm CuO NPs after three weeks. Bar = 200 μm.

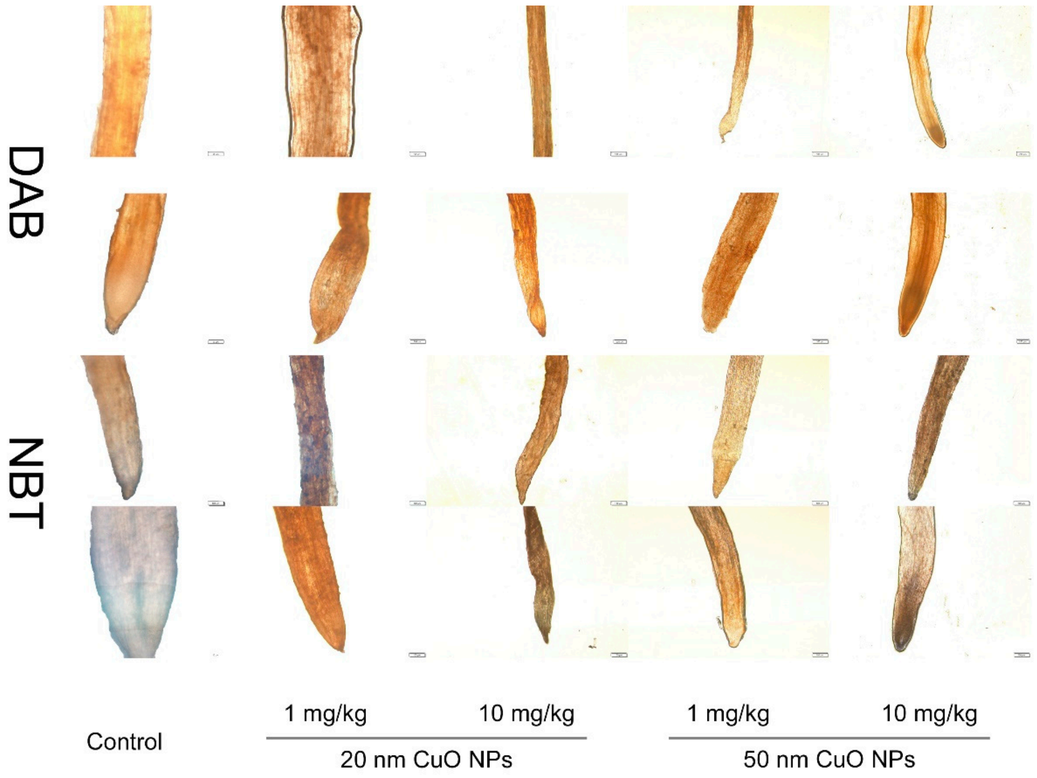

Figure S3 DAB and NBT staining of root tips under the treatments of 1 and 10 mg/kg of 20 and 50 nm CuO NPs after three weeks. Bar = 100 μm.

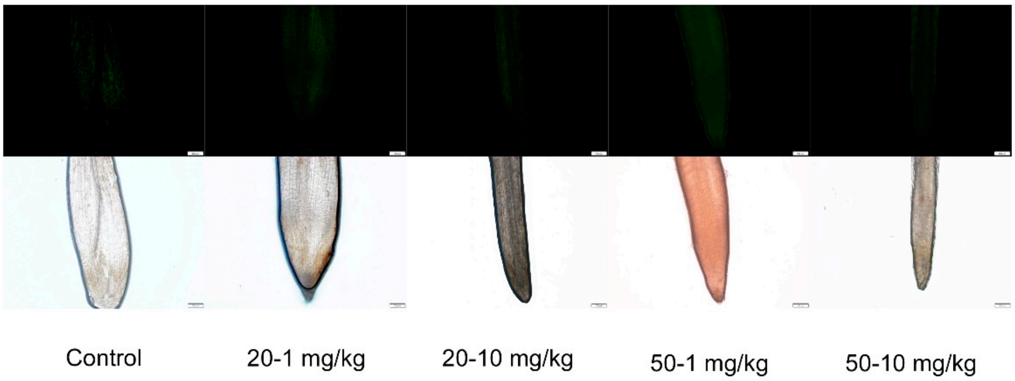

Figure S4 DCFH-DA staining of soybean root under the treatments of 1 and 10 mg/kg of 20 and 50 nm CuO NPs after three weeks. Bar = 100 μm.

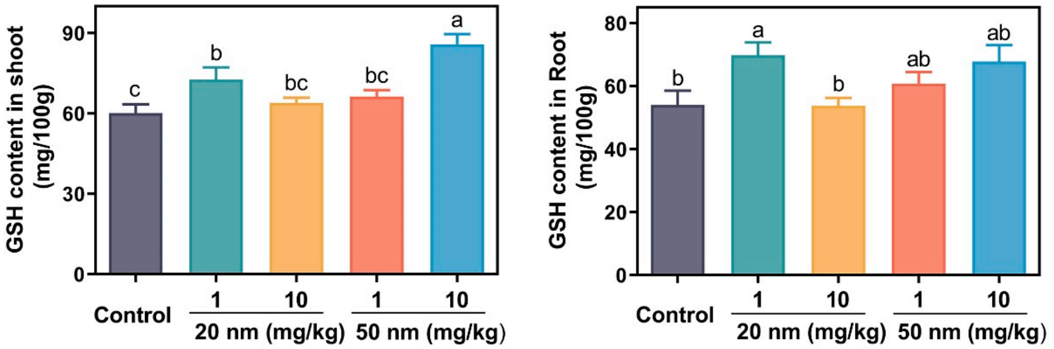

Figure S5 GSH content in soybean shoot and root under the treatment of 1 and 10 mg/kg of 20 and 50 nm CuO NPs after three weeks. Error bars represent standard error of 5 replicates, and different letters in each panel were significantly different at $p < 0.05$.

Table S1. Length, fresh weight and dry weight of soybean plants under the treatment of 20 nm CuO NPs in pre-experiment.

Treatments	Shoot			Root		
	Length / cm	Fresh weight / g	Dry weight / g	Length / cm	Fresh weight / g	Dry weight / g
Control	38.8 ± 7.3	3.5 ± 0.3	0.58 ± 0.03	17.0 ± 2.1	1.9 ± 0.3	0.15 ± 0.01
1 mg/kg	45.8 ± 9.0	4.1 ± 0.5	0.70 ± 0.11	16.7 ± 2.2	2.6 ± 0.8	0.24 ± 0.04
5 mg/kg	43.6 ± 7.6	3.7 ± 0.4	0.63 ± 0.07	17.3 ± 2.3	2.0 ± 0.3	0.17 ± 0.02
10 mg/kg	48.2 ± 9.8	4.0 ± 0.5	0.69 ± 0.12	16.1 ± 1.0	2.1 ± 0.4	0.18 ± 0.03
25 mg/kg	48.2 ± 8.2	3.9 ± 0.6	0.69 ± 0.11	15.9 ± 1.4	1.8 ± 0.4	0.16 ± 0.02
50 mg/kg	46.0 ± 7.1	3.9 ± 0.6	0.69 ± 0.13	17.7 ± 3.0	2.0 ± 0.4	0.16 ± 0.02

Note: Value are means of 5 plants ± SD. The one-way analysis of variance showed no significant difference across all the treatments.

Table S2. Length and fresh weight and dry weight of soybean plants under the treatment of 50 nm CuO NPs in pre-experiment.

Treatments	Shoot			Root		
	Length / cm	Fresh weight / g	Dry weight / g	Length / cm	Fresh weight / g	Dry weight / g
Control	32.0 ± 6.4	3.5 ± 0.7	0.60 ± 0.05	18.4 ± 2.4	2.1 ± 0.2	0.16 ±0.03
1 mg/kg	37.7 ± 4.0	4.0 ± 0.5	0.70 ± 0.12	17.6 ± 2.3	2.5 ± 0.5	0.19 ± 0.04
5 mg/kg	34.6 ± 4.4	3.8 ± 0.4	0.70 ± 0.07	16.4 ± 0.9	2.2 ± 0.5	0.17 ± 0.03
10 mg/kg	38.2 ± 7.2	3.5 ± 0.6	0.64 ± 0.10	16.2 ± 1.4	2.0 ± 0.2	0.16 ± 0.03
25 mg/kg	35.0 ± 6.0	3.5 ± 0.4	0.65 ± 0.08	16.0 ± 1.7	2.0 ± 0.3	0.17 ± 0.03
50 mg/kg	33.0 ± 3.7	3.7 ± 0.4	0.69 ± 0.03	17.0 ± 2.6	1.8 ± 0.2	0.16 ± 0.01

Note: Value are means of 5 plants ± SD. The one-way analysis of variance showed no significant difference across all the treatments.

Table S3. Nitrate nitrogen content in shoot and root of soybean plant in pre-experiment.

Treatment	20 nm CuO NPs		50 nm CuO NPs	
	Shoot	Root	Shoot	Root
Control	0.22 ± 0.08	0.18 ± 0.06	0.20 ± 0.09	0.23 ± 0.02
1 mg/kg	0.32 ± 0.11	0.25 ± 0.09	0.19 ± 0.02	0.20 ± 0.07
5 mg/kg	0.29 ± 0.06	0.23 ± 0.13	0.20 ± 0.05	0.21 ± 0.07
10 mg/kg	0.28 ± 0.11	0.22 ± 0.02	0.20 ± 0.05	0.18 ± 0.02
25 mg/kg	0.24 ± 0.06	0.16 ± 0.03	0.21 ± 0.06	0.23 ± 0.05
50 mg/kg	0.22 ± 0.07	0.19 ± 0.03	0.19 ± 0.06	0.22 ± 0.01

Note: Value are means of 5 plants ± SD. The one-way analysis of variance showed no significant difference across all the treatments. The unit of Nitrate nitrogen content in the table is milligram per gram dry weight.

Table S4. Shoot length and root length of soybean plant.

Treatments	Shoot length / cm	Root length / cm
Control	56.4 ± 7.7	21.4 ± 1.8
20 nm 1 mg/kg	65.3 ± 7.5	23.8 ± 2.8
20 nm 10 mg/kg	63.6 ± 4.5	21.4 ± 1.4
50 nm 1 mg/kg	65.1 ± 9.0	21.6 ± 3.1
50 nm 10 mg/kg	73.9 ± 3.6	20.9 ± 2.2

Note: Value are means of 8 plants ± SD. The one-way analysis of variance showed no significant difference across all the treatments.

Table S5. Elements content in soybean root, shoot and soil.

		Control	20 nm CuO NPs		50 nm CuO NPs	
			1 mg/kg	10 mg/kg	1 mg/kg	10 mg/kg
Shoot	K	27988 ± 564 [a]	27117 ± 612 [a]	25354 ± 705 [b]	24358 ± 826 [b]	22202 ± 546 [c]
	P	3195 ± 261 [b]	3832 ± 128 [a]	3306 ± 197 [b]	3267 ± 258 [b]	3184 ± 134 [b]
	Mn	111.2 ± 6.3 [b]	133.2 ± 3.7 [a]	104.2 ± 7.4 [b]	55.5 ± 5.5 [c]	100.7 ± 10.9 [b]
	Fe	194.9 ± 16.3 [b]	228.4 ± 16.9 [ab]	253.6 ± 13.2 [a]	199.2 ± 36.5 [b]	226.9 ± 42.5 [ab]
Root	K	32421 ± 2011 [bc]	35555 ± 2071 [ab]	30703 ± 3387 [c]	37312 ± 1747 [a]	39938 ± 4367 [a]
	P	3202 ± 509 [b]	3969 ± 251 [a]	3227 ± 353 [b]	3328 ± 569 [ab]	2774 ± 397 [b]
	Mn	210.4 ± 37.9 [a]	168.0 ± 20.5 [abc]	183.1 ± 36.1 [ab]	154.7 ± 24.2 [bc]	137.0 ±19.4 [c]
	Fe	2270 ± 409 [b]	3155 ± 796 [ab]	2876 ± 661 [ab]	3325 ± 656 [a]	2491 ± 219 [ab]
Soil	K	8676 ± 603 [a]	9060 ± 376 [a]	8977 ± 525 [a]	9762 ± 823 [a]	9222 ± 1205 [a]
	P	1116 ± 60 [b]	1284 ± 56 [b]	1307 ± 105 [b]	1318 ± 100 [b]	1542 ± 266 [ab]
	Mn	557.4 ± 23.2 [a]	514.1 ± 16.2 [b]	500.5 ± 11.0 [b]	513.1 ± 7.4 [b]	541.7 ± 20.6 [a]
	Fe	30883 ± 605 [b]	30751 ± 1161 [b]	30714 ± 721 [b]	32891 ± 959 [ab]	36560 ± 6959 [a]

Note: Value are means of 5 plants ± SD. The different letters superscripted indicate that these parameters were significantly differed between CuO NP treatments and control at $p < 0.05$. The unit of elements content in the table is milligrams per kilogram of dry weight.

References

1. Shaw, A.K.; Ghosh, S.; Kalaji, H.M.; Bosa, K.; Brestic, M.; Zivcak, M.; Hossain, Z. Nano-CuO stress induced modulation of antioxidative defense and photosynthetic performance of Syrian barley (Hordeum vulgare L.). *Environmental and Experimental Botany* **2014**, *102*, 37-47, doi:10.1016/j.envexpbot.2014.02.016.
2. Nguyen, H.M.; Sako, K.; Matsui, A.; Suzuki, Y.; Mostofa, M.G.; Ha, C.V.; Tanaka, M.; Tran, L.P.; Habu, Y.; Seki, M. Ethanol Enhances High-Salinity Stress Tolerance by Detoxifying Reactive Oxygen Species in Arabidopsis thaliana and Rice. *Frontiers in Plant Science* **2017**, *8*, 1001, doi:10.3389/fpls.2017.01001.
3. Wang, S.; Liu, H.; Zhang, Y.; Xin, H. The effect of CuO NPs on reactive oxygen species and cell cycle gene expression in roots of rice. *Environ Toxicol Chem* **2015**, *34*, 554-561, doi:10.1002/etc.2826.
